# Enhancing learning gains through gamification and collaborative learning strategies

**DOI:** 10.3389/fmed.2026.1887898

**Published:** 2026-07-31

**Authors:** Blanca Bazán-Perkins, Valeria Andrea Zuñiga, Andrés Arispe-Oliver, José Alfredo Santibañez-Salgado, Angélica Flores-Flores

**Affiliations:** 1 Tecnologico de Monterrey, Escuela de Medicina y Ciencias de la Salud, Mexico City, Mexico; 2 Laboratorio de Inmunofarmacología, Instituto Nacional de Enfermedades Respiratorias Ismael Cosio Villegas, Mexico City, Mexico; 3 Programa Universitario de Estudios sobre Democracia, Justicia y Sociedad, Universidad Nacional Autónoma de Mexico, Mexico City, Mexico

**Keywords:** collaborative learning, gamification, health sciences, learning gain, motivation

## Abstract

**Introduction:**

Gamification and collaborative learning are key pedagogical strategies that foster student interaction, academic productivity, and motivation. Understanding the biochemical intricacies of cellular metabolism is a challenging endeavor, and the use of metabolic maps makes this task more accessible through these approaches. In this study, we integrated gamification as a teaching strategy to encourage student engagement within a collaborative learning framework while constructing a metabolic map. Our goal was to determine whether this combination was associated with measurable learning gains.

**Methods:**

We conducted a quasi-experimental study with a non-equivalent control group and pretest-posttest measurements, using a convenience sample of students from the School of Medicine and Health Sciences at Tecnológico de Monterrey, Mexico City Campus. Each student first created an individual hand-drawn metabolic map. At the end of the course, they worked together to produce a collaborative map that incorporated gamification and collaborative learning elements. Participants completed questionnaires assessing normalized learning gains and situational motivation. Data were analyzed using descriptive statistics and Pearson correlation coefficients.

**Results:**

Students reported that both individual and group maps offered distinct learning benefits. They particularly noted that the collaborative process deepened their analysis of metabolic pathways. The integration of gamification and collaborative learning was associated with significant improvements in normalized learning gains, intrinsic motivation, identified extrinsic regulation, and the self-determination index. Furthermore, the magnitude of normalized learning gains was positively correlated with intrinsic motivation, identified extrinsic regulation, and the self-determination index.

**Conclusion:**

In summary, the results of this study suggest that implementing gamification and collaborative learning environments is associated with increases in intrinsic motivation, identified extrinsic motivation, and the self-determination index, which in turn correlate with improved learning gains. These findings indicate that such strategies may effectively strengthen educational programs and boost academic performance, although further research with more rigorous designs is warranted to establish causal relationships.

## 1 Introduction

Collaborative learning serves as a valuable educational approach that promotes student interaction, supports productivity, and contributes to both individual and collective performance (1). Within such collaborative settings, group work has been associated with intrinsic motivation (2). Motivation is a fundamental dimension of human experience that encompasses intrinsic motivation, extrinsic motivation, and amotivation. According to self-determination theory, motivation ranges from intrinsic—autonomous, spontaneous, voluntary, and internally driven—to controlled, which is externally driven by outside pressures. Extrinsic motivation itself exists on a spectrum, from external regulation (the least self-determined form) to integrated regulation (the most self-determined) (3). At the lowest end of self-determination lies amotivation, characterized by a perceived disconnect between actions and outcomes, leading to feelings of incompetence and a complete lack of drive. Both intrinsic and extrinsic motivation are dynamic states regulated by self-regulation and self-determination processes, and they are shaped by personal experiences (3, 4).

Gamification is an instructional strategy that applies game-design elements to non-game contexts. It is also recognized for its capacity to generate motivational conditions by creating environments that promote autonomy, self-affirmation, competence, and task relevance, thereby supporting learning gains (5, 6). The term “learning gain” broadly refers to measurable improvements in learning resulting from a specific educational intervention. Assessing the level of learning achieved can help determine whether a particular instructional strategy or style is more effective (7–9).

Teaching cellular metabolism presents a significant pedagogical challenge due to the complexity of its biochemical reactions, the wide variety of molecules involved, and the intricate regulation of each metabolic pathway (10). A range of strategies has been proposed to facilitate the study of metabolism, including videos (11), animations (12), origami-based models (13) and metabolic maps remains a widely adopted approach (14, 15).

Despite the recognized benefits of collaborative learning and gamification as independent strategies (1, 5), no studies to date have examined their combined effect on both learning gains and motivational outcomes in the context of metabolic map construction. Given the pedagogical challenges posed by teaching cellular metabolism (10), there is a clear need for innovative instructional approaches that simultaneously support knowledge acquisition and student engagement.

Therefore, the present study aims to fill this gap by investigating the learning gains and motivational changes associated with a collaborative and gamified approach to metabolic map construction among medical and health sciences students. We hypothesize that this combined strategy will be associated with significant improvements in both learning and adaptive motivation, providing empirical evidence for the integration of active methodologies in basic science education.

## 2 Materials and methods

### 2.1 Study design and participants

**Figure 1:**
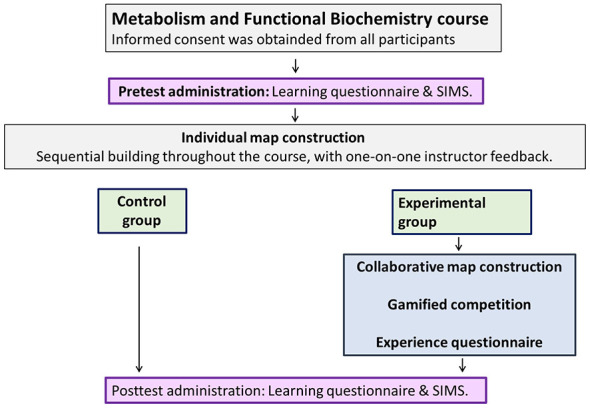
Study design flowchart. The diagram shows the quasi-experimental design comparing the collaborative-gamified map (Experimental group) vs. the individual map only (Control group). Both groups shared an identical baseline (individual map construction with instructor feedback) and completed the same pretest and posttest (learning questionnaire and Situational Motivation Scale, SIMS). The intervention in the experimental group involved team-based collaborative poster creation and a gamified competition. Comparability is ensured by the shared baseline, equivalent content and time, and identical outcome measures, thus isolating the effect of the collaborative-gamified methodology.

A quasi-experimental design with a non-equivalent control group and pretest-posttest measurements was employed to evaluate the impact of collaborative learning and gamification on academic performance and motivation (Figure 1). First-semester students enrolled in the Metabolism and Functional Biochemistry course at Tecnológico de Monterrey (Mexico City Campus) were invited to participate. The sample included students from the Medical Surgeon, Nutrition, and Biomedical Engineering programs.

Eligibility criteria included completion of the course, submission of all questionnaires, and provision of informed consent. Data handling complied with Mexican privacy regulations (INER S01-16). Allocation to groups was not randomized; rather, it was determined by pre-existing class groups formed through the university's official enrollment process. Assignment of class groups to conditions was based on logistical considerations, including classroom availability and the academic timetable, and was not influenced by student demographics or prior academic performance. This approach ensured that all members of a given class group experienced the same condition, thereby minimizing the risk of intra-group contamination.

### 2.2 Procedure and intervention

#### 2.2.1 Individual metabolic maps

At the start of the course, each student began constructing a hand-drawn metabolic map integrating the major catabolic and anabolic pathways of carbohydrates, lipids, amino acids, and nucleic acids. However, rather than completing the entire map in a single session, the work was divided into sequential parts. After each class session, students received specific instructions on which segment of the map to develop for the following meeting. This progressive approach allowed students to build their maps incrementally, incorporating new content as it was covered in class. The task followed the detailed criteria outlined in Supplementary File 1, which specified biochemical depth (structures, enzymes, and cofactors), pathway interconnections, organelle-specific localization, and the rule that metabolites could only appear in multiple compartments if functionally justified.

At the beginning of each subsequent class, the instructor reviewed the corresponding part of each student's map. During this one-on-one feedback session, the instructor pointed out inaccuracies, suggested corrections, and discussed why specific segments were correctly or incorrectly represented. This iterative review process ensured that students received continuous guidance and could correct mistakes before moving on to the next section. Grading at this stage was not based on achieving a fully correct map from the outset, since complete and accurate integration was rarely attained in the early phases. Instead, the evaluation focused on the student's genuine attempt to follow the prescribed structure for each part—assessing whether the required pathways were included, whether the organizational layout matched the compartmentalization guidelines, and whether the student had made a reasonable effort to connect the routes as instructed for that specific segment. During each review, the instructor explained the rationale behind the grade, reinforcing why certain parts were acceptable and where improvements were needed.

**Figure 2:**
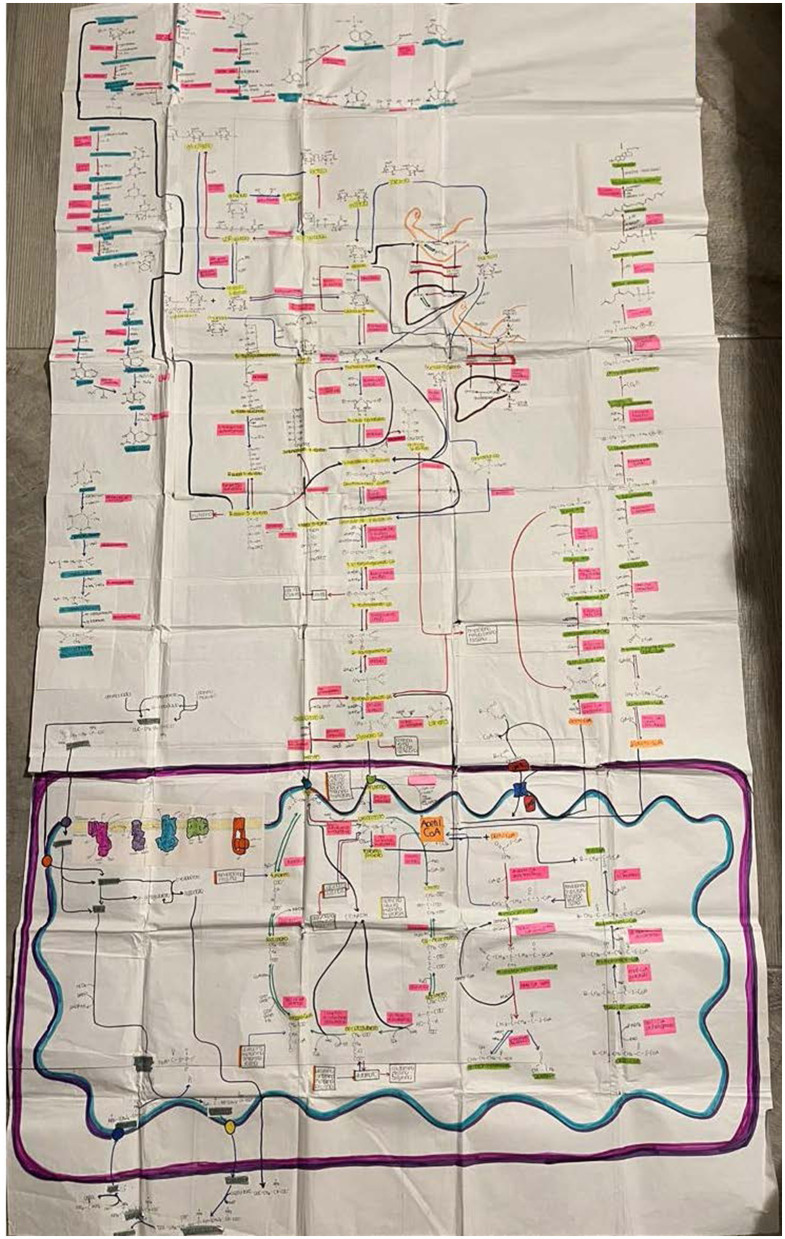
Example of an individual hand-drawn metabolic map. This map illustrates the integration of major catabolic and anabolic pathways of carbohydrates, lipids, amino acids, and nucleic acids, following the guidelines specified in Supplementary File 1. Students constructed these maps incrementally over the course, receiving iterative feedback from instructors after each segment. This example represents a completed individual map assembled at the end of the sequential construction process, prior to the collaborative intervention. Image courtesy of Brenda Meza (student), reproduced with permission.

Once all individual parts were completed and reviewed, the final assembled map served as a baseline to assess learning progress following a collaborative and gamified intervention (Figure 2), allowing a direct comparison between initial independent performance and subsequent group-based improvement.

#### 2.2.2 Collaborative maps and gamified competition

**Figure 3:**
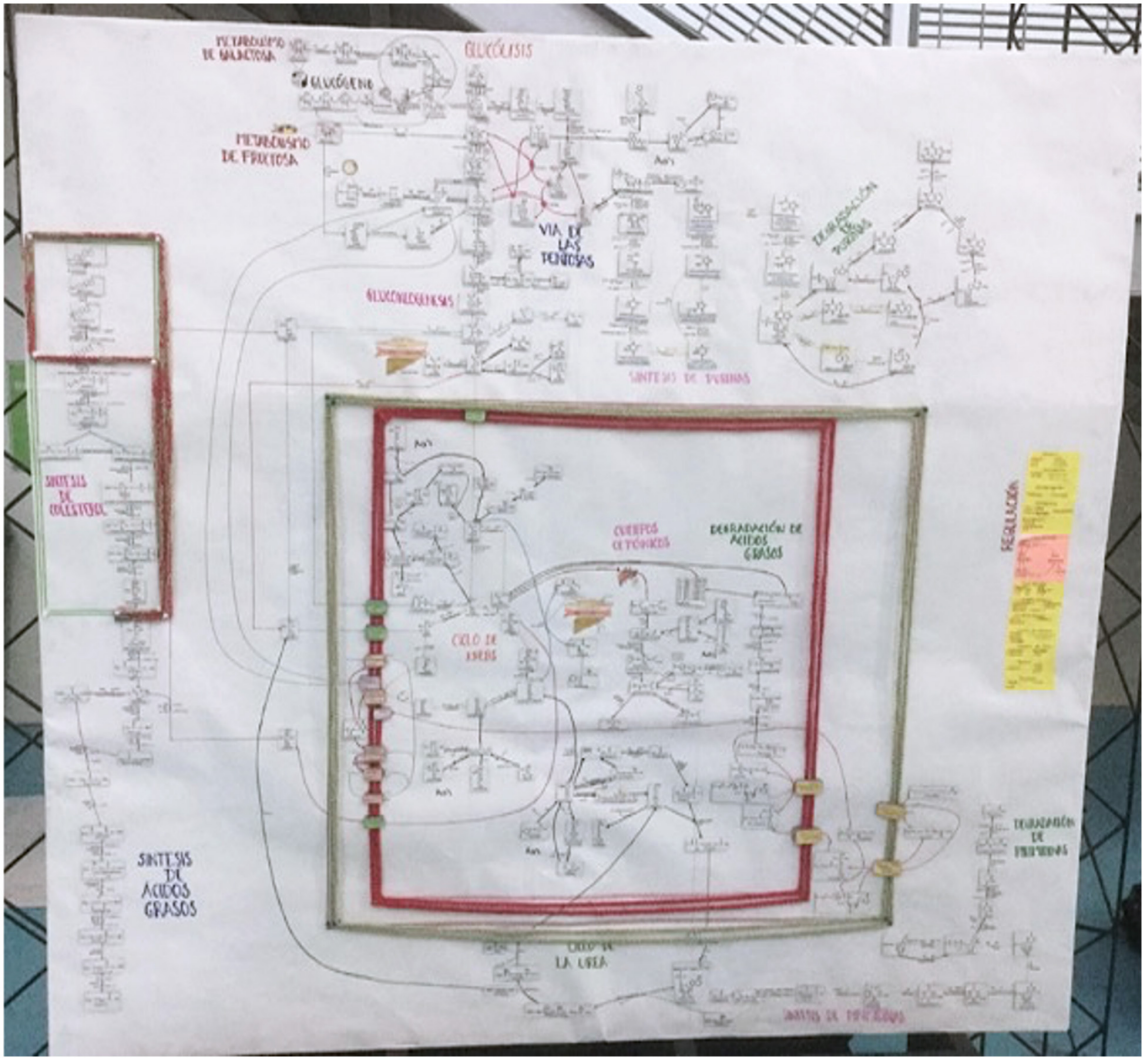
Example of a collaborative metabolic map in poster format. This poster was produced by a three-member team as part of the gamified competition, following the prescribed size and design specifications (Supplementary File 2). The collaborative map integrates the major catabolic and anabolic pathways of carbohydrates, lipids, amino acids, and nucleic acids, reflecting the team's negotiated decisions on pathway interconnections, organelle-specific localization, and biochemical detail (structures, enzymes, and cofactors). This example illustrates the outcome of peer discussion and consensus-building among team members with different academic backgrounds and individual mapping approaches. Image courtesy of Arely Elizabeth Nataren, Mayra Perez, and Eduardo Sayavedra (students), reproduced with permission.

Following the cluster-randomized assignment of class groups to the experimental condition, students within these groups were organized into three-member teams. Each team was deliberately composed of high- and low-performing students drawn from different academic programs to foster peer learning and encourage exposure to diverse analytical approaches (16). Teams were instructed to produce a collaborative metabolic map in poster format, following the prescribed size and design specifications (Supplementary File 2; Figure 3). Because students had already gained hands-on experience through the sequential construction of their individual metabolic maps, the team-based poster creation became a valuable opportunity for exchanging ideas and negotiating how best to integrate each pathway component. However, since each student had developed their own approach to organizing and representing the pathways, discrepancies frequently arose within teams regarding the most appropriate way to introduce or connect a given route. During this stage, it was common for teams to seek instructor support to help resolve these disagreements, particularly when members had adopted substantially different strategies in their individual maps.

The collaborative project was implemented as a gamified competition. Each team's poster was assessed by pairs of volunteer advanced students using a two-component rubric (Supplementary File 3), which evaluated: (a) visual and organizational attributes, including clarity, creativity, and layout; and (b) the completeness of content across all metabolic pathways. The evaluation process was organized into sequential rounds, with 10 posters being reviewed during each 30-min session. In each round, the 10 posters were simultaneously assessed by two evaluator teams, generating four independent scores per poster. Upon completion of each round, the subsequent cohort of 10 posters was installed for evaluation. Before posters were removed, teams received up to two ribbon-based badges acknowledging specific qualities of their work, as recommended by the evaluators, and were informed of the highest average scores achieved. Nevertheless, eligibility for the finalist phase was only established once all rounds had been concluded.

The top 10 scoring teams qualified for a final competition, in which faculty professors examined them on hormonal regulation (insulin, glucagon, cortisol, and epinephrine) of specific pathways. Students were required to explain intervention points and metabolic effects, thereby assessing higher-order conceptual understanding beyond the visual representation (Supplementary File 4). The top three teams received diplomas awarded by the Dean of the School of Medicine.

### 2.3 Instruments

#### 2.3.1 Learning gain questionnaire

A 40-item true/false questionnaire was developed to assess students' knowledge of key concepts in metabolic pathways. Content validity was established through expert review by three experienced biochemistry faculty members, who evaluated each item for accuracy, clarity, and alignment with the course learning objectives. The complete questionnaire is provided as Supplementary File 5. We acknowledge that true/false questions involve a 50% chance of guessing; however, we followed the standard interpretation in the field, where a normalized learning gain ≥ 0.3 indicates meaningful learning (17, 18). Each student's normalized learning gain was calculated using Hake's formula:


$$\begin{array}{r} \text{Normalized learning gain}=\\ \left(\text{Posttest}-\text{Pretest}\right)/\left(\text{Max score}-\text{Pretest}\right) \end{array}$$

where the Pre-test refers to the score from the initial questionnaire and the Post-test to the score from the end-of-course questionnaire. Since the questionnaire contained 40 items, scores were expressed as percentages for the calculation. This questionnaire was completed in the classroom before the course started and again during the last class before the final exam (scheduled a few days later), with the post-test timing chosen to discourage students from studying specifically for it.

#### 2.3.2 Situational Motivation Scale

Motivation was assessed using the Situational Motivation Scale (SIMS). Participants rated their agreement with various statements on a 7-point Likert scale (1 = “strongly disagree” to 7 = “strongly agree”). The SIMS includes four subscales, each measuring a distinct motivational dimension with four items: intrinsic motivation (IM), identified regulation (IR), external regulation (ER), and amotivation (A). A Self-Determination Index (SDI) was calculated using the following formula:


$$\begin{array}{l} \text{Self}-\text{determination index}=\left(2\times \text{IM}\right)+\text{IR}-\text{ER}-\left(2\times \text{A}\right) \end{array}$$

Previous research has confirmed the validity and reliability of the SIMS in both English and Spanish across multiple disciplines (19–21). Both the learning gain questionnaire and the SIMS have been previously used in studies with medical students (22, 23).

#### 2.3.3 Experience of both metabolic maps construction

At the end of the course, students who had completed both the individual and collaborative metabolic maps were surveyed about their learning experience. The survey consisted of two questions. The first question asked:

“Do you prefer working on the individual map alone or both maps (individual + collaborative)?”

with two response options: “individual only” or “both maps.” The second question was open-ended: “Why?” allowing students to elaborate on their preference.

For the analysis, responses to the first question were examined using simple frequency counts, as the data were categorical with two mutually exclusive options. For the open-ended responses, two researchers independently reviewed the answers to identify recurring themes. After independent review, both researchers reached consensus on the final thematic categories. The results presented in the manuscript reflect the most frequently reported reasons, which were then reinterpreted and summarized in the findings section.

### 2.4 Data collection

After agreeing to participate, students were randomly assigned an identification number to use when completing all questionnaires. The SIMS and learning gain questionnaires were administered during regular class hours. Students were given 40 min to answer the 40 learning gain questions and 10 min for the SIMS. An additional questionnaire about students' experience working with both individual and collaborative maps was administered only to students who had participated in both conditions and was completed outside of class at the end of the course.

The cover page of each questionnaire explained the study's purpose, stated that participation was voluntary, and assured participants that the collected data would remain anonymous throughout the study.

### 2.5 Statistical analysis

Data were organized using MS Office Excel 2016 (Microsoft Corporation, Redmond, Washington, USA). Statistical analyses were conducted with STATA 17 (StataCorp, College Station, TX, USA). Normality was assessed using the Shapiro–Wilk test, and homogeneity of variances was verified with Levene's test. Baseline demographic differences between groups were analyzed using independent-samples t-tests (age) and chi-square tests (sex distribution). For within-group comparisons (pre-test vs. post-test), paired-samples t-tests were conducted, while independent-samples *t*-tests were used for between-group comparisons. To control for multiple comparisons across the SIMS subscales (intrinsic motivation, identified extrinsic motivation, external extrinsic motivation, amotivation, and self-determination index, pre- and post-intervention), a Bonferroni correction was applied, setting the significance threshold at *p* < 0.008 (0.05/6). Effect sizes (Cohen's *d*) with 95% confidence intervals were calculated for all significant comparisons, using pre-test SD for paired comparisons and pooled SD for independent comparisons. Effect sizes were interpreted according to Cohen's (24) conventions: small (*d* ≥ 0.20), medium (*d* ≥ 0.50), and large (*d* ≥ 0.80). The relationship between changes in motivational variables (Δ = post-test – pre-test) and normalized learning gain was examined using Pearson correlation coefficients. Statistical significance was set at *p* < 0.05, and all tests were two-tailed.

## 3 Results

### 3.1 Participant demographics

A total of 98 questionnaires were initially collected; however, only 85 were fully and correctly completed and thus included in the final analysis. The control group comprised 40 students (69% female, mean age = 19.3 ± 1.5 years), while the experimental group consisted of 45 students (71% female, mean age = 19.5 ± 1.2 years). Independent-samples *t*-tests and chi-square tests revealed no significant differences between groups in age [*t*(83) = −0.67, *p* = 0.505] or sex distribution [χ^2^(1) = 0.04, *p* = 0.842), indicating that the two groups were equivalent with respect to these demographic variables at the outset of the study.

### 3.2 Experience of metabolic maps construction

The metabolic maps produced varied considerably in both size and visual style. During the construction of individual maps, students frequently expressed doubts and required ongoing guidance from instructors. In contrast, when working collaboratively on maps at the end of the course, students produced better-constructed maps and requested assistance far less often.

Students who had completed both individual and collaborative maps were surveyed about their experiences. When asked to choose between creating only one type of map, the vast majority (78%) responded that they would prefer to do both. They provided several reasons for this preference. The individual map, which they described as the more challenging of the two, forced them to thoroughly learn the structure and name of each molecule as well as each chemical reaction. The collaborative map, on the other hand, required them to engage in reasoning and analysis of metabolic processes. Students also felt that the map contest was valuable because it motivated them not only to invest effort in constructing the map but also to study together as a team in order to answer the evaluators' questions and win the competition.

### 3.3 Impact of collaborative learning and gamification on learning gains and motivation

The combination of gamification and collaborative learning was associated with a significant increase in normalized learning gain compared to the control group (*p* < 0.001; Table 1). The experimental group demonstrated substantially higher learning gains, with a large effect size [*d* = 2.22, 95% CI (1.68, 2.76)], indicating a practically meaningful improvement in academic performance.

**Table 1:** Normalized learning gain and situational motivation scale pre- and post-intervention in collaborative learning and gamification environments.

Assessment dimension		Control (*n* = 40)		Gamification and collaborative learning (*n* = 45)		Cohen's *d* [IC 95%]
	Normalized learning gain	0.49 ± 0.11		0.69 ± 0.068^**&**^		2.22 [1.68, 2.76]
		*Pre-*	*Post-*	*Pre-*	*Post-*	
Situational motivation scale	Intrinsic motivation	4.18 ± 1.42	3.85 ± 1.75	4.69 ± 1.11	5.66 ± 1.15^*^p = 1.2 × 10^−15^	0.87 [0.53, 1.22]
	Extrinsic motivation identified	4.20 ± 1.41	4.54 ± 2.11	4.70 ± 1.29	5.87 ± 0.95^*^p = 1.2 × 10^−15^	0.91 [0.56, 1.25]
	Extrinsic motivation external	5.10 ± 1.18	5.00 ± 1.45	4.95 ± 0.89	5.06 ± 0.82	–
	Amotivation	4.39 ± 2.11	3.83 ± 1.15	3.94 ± 0.95	3.76 ± 0.78	–
	Self-determination index	−0.18 ± 0.93	−0.16 ± 0.99	−0.47 ± 1.04	1.85 ± 1.07^*^p = 1.2 × 10^−15^	2.23 [1.69, 2.78]

Values correspond to mean ± SD; ^&^unpaired Student's t-test, ^*^paired Student's t-test. A Bonferroni correction was applied for multiple comparisons across SIMS subscales. The corrected significance level was set at p < 0.008 (0.05/6). All comparisons marked with ^*^ or ^&^ remained significant after correction. Effect sizes (Cohen's *d*) are reported for significant comparisons.

Results from the SIMS questionnaire revealed that the incorporation of collaborative learning and gamification was associated with significant increases in intrinsic motivation, identified extrinsic motivation, and the self-determination index when comparing pre- and post-intervention SIMS scores within the experimental group (*p* < 0.001 for all comparisons; Table 1).

Baseline SIMS values showed no significant differences between the control group and the experimental group, confirming that both groups were equivalent in terms of motivation at the start of the course. In contrast, no significant changes were observed in the control group for these variables. Cohen's *d* effect sizes with 95% confidence intervals were calculated for all significant comparisons. Large effects were observed for the self-determination index [*d* = 2.23, 95% CI (1.69, 2.78)], intrinsic motivation [*d* = 0.87, 95% CI (0.53, 1.22)], and identified extrinsic motivation [*d* = 0.91, 95% CI (0.56, 1.25)]. All confidence intervals exceeded the threshold for a large effect (*d* > 0.80), confirming that the observed differences were practically meaningful beyond statistical significance.

### 3.4 Correlation between motivation and learning gains

**Figure 4:**
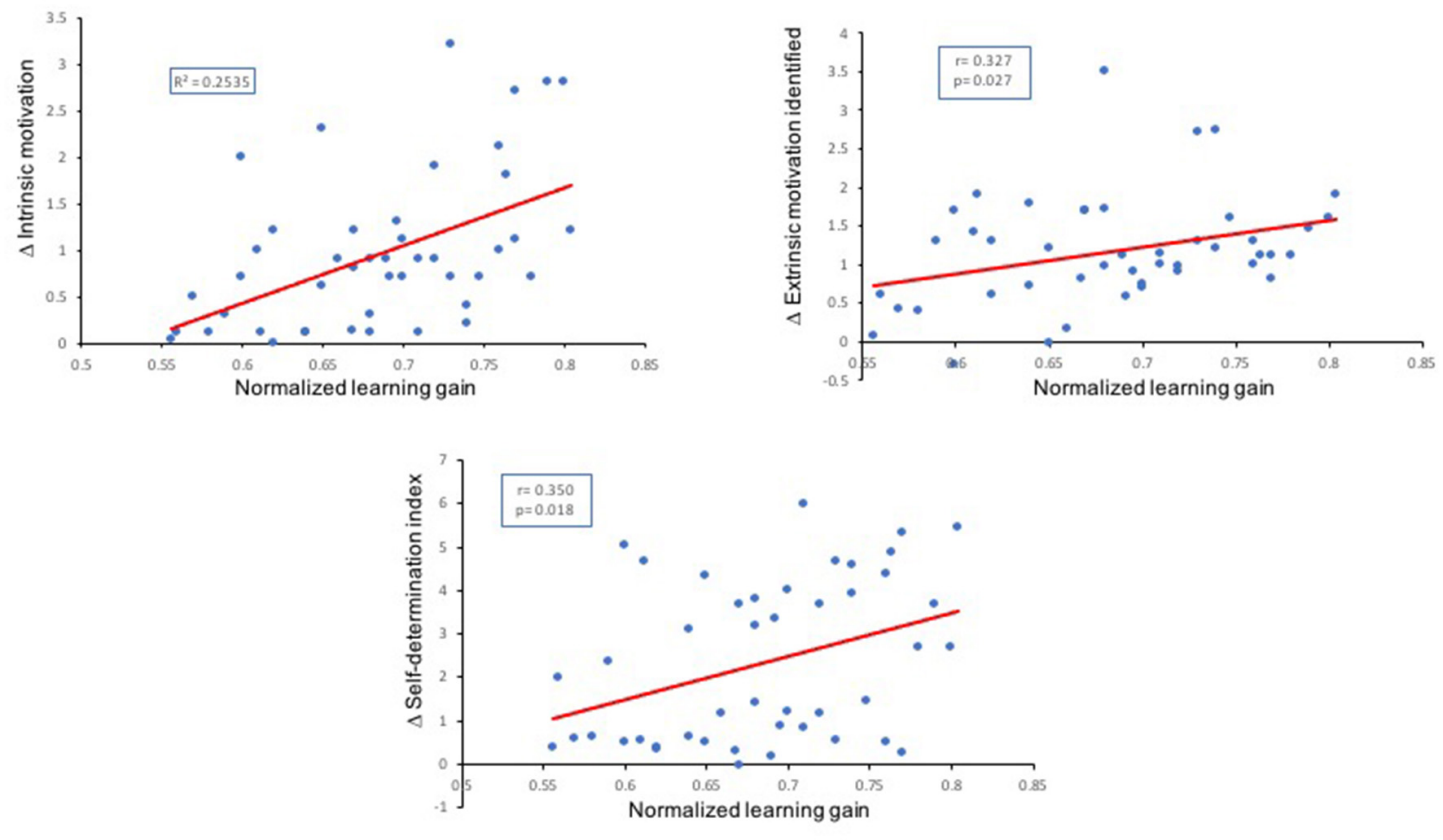
Relationship between motivation and normalized learning gain. Scatter plots showing that the difference between post- and pre- (Δ) intrinsic motivation, external identified regulation, and self-determination index correlated with the magnitude of normalized learning gain. *r* = Pearson correlation coefficient.

A significant positive correlation was observed between the magnitude of change (Δ) in intrinsic motivation (*r* = 0.503; *R*^2^ = 0.253; *p* = 0.00042), extrinsic motivation (*r* = 0.32; *R*^2^ = 0.107; *p* = 0.027), and the self-determination index (*r* = 0.35; *R*^2^ = 0.122; *p* = 0.018) and the level of normalized learning gain (Figure 4). This suggests that students who experienced greater motivational improvements also achieved learning gains, supporting the theoretical link between motivation, mainly intrinsic motivation, and academic performance in the context of gamified collaborative learning.

## 4 Discussion

### 4.1 Overview of main findings

This study examined whether integrating gamification and collaborative learning into the construction of metabolic maps was associated with enhanced student motivation and academic performance in a first-year health sciences biochemistry course. Our findings indicate that the combination of these strategies was associated with significant improvements in both learning gains and motivational outcomes, with large effect sizes that suggest practical meaningfulness of the intervention.

### 4.2 Motivational outcomes and self-determination theory

The significant increases in intrinsic motivation, identified extrinsic motivation, and the self-determination index observed in the experimental group align with the theoretical framework of self-determination theory (25). Gamification components function as persuasive strategies to drive behavioral change (26), addressing the basic psychological needs for competence (through challenges, feedback, and badges), autonomy (through choice and self-regulation), and relatedness (through social comparison and team competition). Collaborative learning, likewise, serves as part of these persuasive strategies, motivating desired behaviors, attitudes, or decisions through rewards, social comparison, competition, and social learning (26, 27). The combination of both strategies was associated with a rise in intrinsic motivation, which plays a key role in the teaching-learning process by fostering enjoyment, conceptual understanding, and the development of meaningful learning (28). This approach was also related to enhanced identified extrinsic motivation, characterized by the student's personal endorsement of goals, where they recognize the importance of a behavior and accept its regulation as their own (25).

### 4.3 Correlation between motivation and academic performance

The significant positive correlations between changes in intrinsic motivation, identified extrinsic motivation, and the self-determination index with normalized learning gains provide empirical support for the theoretical link between motivation and academic performance. Notably, intrinsic motivation showed the strongest correlation, consistent with previous research indicating that intrinsically motivated students engage more deeply with content, persist longer in challenging tasks, and achieve better learning outcomes (25, 28). These findings are consistent with a systematic review and meta-analysis reporting that gamification enhances engagement in computerized cognitive training (29). Similarly, in medical education, gamification has been shown to function as an effective active learning strategy that increases engagement, promotes teamwork, provides immediate feedback, and renders content more appealing (30).

### 4.4 Complementary roles of individual and collaborative maps

The survey responses provide valuable qualitative insight into the learning process. Students reported that individual maps were challenging but forced them to thoroughly learn molecular structures and reactions, while collaborative maps required reasoning and analysis of metabolic processes. This distinction is noteworthy: individual construction appears to support factual knowledge acquisition, whereas collaborative work promotes higher-order thinking and integration. The finding that 78% of students preferred to do both types of maps suggests that these approaches serve complementary roles in learning, with individual maps building foundational knowledge and collaborative maps fostering deeper conceptual understanding. Students also valued the gamified competition, which motivated them not only to invest effort in constructing the map but also to study together as a team to answer evaluators' questions and win the competition. This supports previous work by Moin et al. (31), who reported that collaborative gamified strategies hold promise as a valuable supplement to traditional instruction in medical education, enhancing engagement, critical thinking, teamwork, and academic performance. The deliberate formation of heterogeneous teams, combining high- and low-performing students, was based on evidence that collaborative dyadic learning in biochemistry leads to significantly better learning outcomes and increased motivation compared to individual study, with both members benefiting from the collaboration through higher motivation, interest, and time spent on task (16).

Our findings extend previous research in several ways. First, while gamification has been studied in various educational contexts, including nursing education where a recent systematic mapping review of 228 studies identified that research on collaborative work and gamification remains underrepresented, with technology-related studies dominating the field (32), our study contributes evidence specifically in the context of metabolic biochemistry—a challenging subject that requires integration of multiple pathways. Second, the large effect sizes observed (*d* = 2.22 for learning gains, *d* = 2.23 for self-determination index) are notably higher than those typically reported in gamification research, suggesting that the combination of collaborative learning and gamification may be associated with synergistic effects. However, these exceptionally large magnitudes warrant a cautious interpretation. Given the quasi-experimental design, the non-randomized allocation of participants, and the simultaneous implementation of multiple pedagogical components—including collaborative work, competition, peer interaction, external recognition, and gamification elements—it is plausible that part of the observed effect size reflects contextual or design-related factors rather than the isolated educational impact of the intervention itself. While the values may be mathematically accurate, they should not be interpreted as unequivocal evidence of exceptional effectiveness in absolute terms, but rather as indicative of strong positive outcomes within the specific conditions of this study. We therefore encourage readers to consider these effect sizes as promising yet preliminary, and to recognize that the magnitude may be influenced by the particular configuration of strategies and the local educational environment. Third, the study design allowed for a direct comparison between individual and collaborative map construction, revealing that these approaches address different aspects of learning: factual knowledge vs. integrative reasoning.

### 4.5 Limitations

While these results are promising, several limitations must be considered when interpreting our findings. From a methodological and design perspective, the quasi-experimental approach with non-random assignment to pre-existing class groups introduces the risk of selection bias, despite the absence of significant baseline differences between groups. The single-institution setting, convenience sampling, and implementation by the course instructors themselves may have introduced unintentional bias and limit the generalizability of our findings to other educational contexts. Regarding measurement and instrumentation, the learning gain questionnaire relies on true/false items that inherently involve a 50% probability of guessing, potentially overestimating actual knowledge acquisition. Although we followed standard practice by applying Hake's normalized gain formula and the instrument was reviewed by experts to establish content validity, we acknowledge that only evidence of content validity is currently available. The questionnaire was not pilot-tested prior to the study, which represents an additional limitation. Future studies should undertake a more comprehensive psychometric validation, including assessments of construct validity, reliability, and item discrimination, to strengthen the robustness of the instrument. Additionally, our motivational outcomes are based exclusively on self-reported measures through the SIMS, which may be subject to social desirability bias and do not capture behavioral or physiological indicators of motivation. Concerning attribution and causal inference, the intervention simultaneously incorporated multiple components—collaborative work, competition, peer interaction, external recognition, and gamification elements—making it impossible to determine the independent contribution of each factor to the observed outcomes. Similarly, while we have included representative examples of individual and collaborative maps along with the assessment rubric in the Supplementary material, the Normalized Learning Gain measures overall learning rather than directly assessing map quality or conceptual reasoning; therefore, conclusions about enhanced pathway analysis should be interpreted with caution. Finally, regarding sample representativeness, the questionnaire assessing students' experience with individual and collaborative maps was administered exclusively to students who participated in both conditions and was completed outside of class time, which may have introduced self-selection bias. A limitation of this study is that the reduced need for instructor guidance during collaborative work, although consistently observed by all faculty members, was not quantitatively measured. While all eligible students completed the questionnaire (100% response rate), it was completed outside of class time, which may have introduced self-selection bias, and students with more positive experiences may have been more likely to respond, potentially skewing the reported perceptions. Likewise, while teams were formed by pairing high- and low-performing students from different programs to encourage peer learning, this introduces heterogeneity in disciplinary background that could influence group dynamics, although all students share a common first-year core curriculum.

### 4.6 Future research directions

Future research should address the aforementioned limitations by employing randomized controlled designs, incorporating objective measures of motivation and reasoning quality, utilizing more robust assessment formats, and exploring the generalizability of these findings across diverse institutional settings and student populations. Additionally, factorial or component-analysis designs would be valuable to disentangle the independent contributions of gamification vs. collaborative learning to the observed outcomes. Future studies could also incorporate systematic rubric-based analysis of individual and collaborative map quality to provide direct evidence of improvements in conceptual reasoning and pathway integration.

## Conclusions

In conclusion, this study provides evidence that integrating gamification and collaborative learning into the construction of metabolic maps is associated with enhanced student motivation and academic performance in medical education. The observed improvements in intrinsic motivation, identified extrinsic motivation, and the self-determination index, along with their positive correlations with learning gains, support the theoretical framework grounded in self-determination theory. While these findings are encouraging, they should be interpreted with caution given the quasi-experimental design and the multifactorial nature of the intervention. Further research with more rigorous designs is needed to establish causal relationships.

## Data Availability

The raw data supporting the conclusions of this article will be made available by the authors, without undue reservation.
