# Supplementary material for: Enhancing learning gains through gamification and collaborative learning strategies
Source: Front Med (Lausanne). 2026 Jul 31;13:1887898. doi: 10.3389/fmed.2026.1887898 (PMC13474011; doi:10.3389/fmed.2026.1887898)
Supplement: Supplementary file 1 [file Table_1.docx]

Supplementary file 1

**Metabolic Map – Official Requirements**

Throughout the course, you will be reviewing various metabolic pathways and processes. As you go through this material, you will gradually build metabolic map that connects and integrates the information you have learned. Your professor will provide specific guidance between classes on which pathways or processes to include at each stage.

The instructions for creating the map are as follows:

1. Hand-Drawn Only

The map must be drawn by hand. Digitally created or computer-generated maps will not be accepted.

2. Mandatory In-Class Submission

You must bring your completed map to class. Do not come to class without it.

3. No In-Class Work

Any student found working on their map during class time will receive a zero for this assignment.

4. Complete Biochemical Detail

The map must include:

The structural formulas (chemical structures) and names of all substrates.

The names of all enzymes, cofactors, coenzymes, and any other molecules involved in each process. For every molecule you draw, you must write its name directly underneath its corresponding structural formula. This applies to all substrates throughout the entire map.

5. Connect All Pathways!

All metabolic routes must be interconnected. Show how they link to one another—do not leave pathways isolated.

6. Simplified Representations Allowed for Certain Pathways

For the following pathways, you do not need to draw molecular structures. Instead, use simple drawings, icons, or diagrams:

Cori Cycle

Glucose-Alanine Cycle

Non-oxidative phase of the Pentose Phosphate Pathway

Oxidative Phosphorylation

Cholesterol Synthesis

7. Structural Detail for Glycogen Metabolism – First Step Only

For the first step of both glycogenesis and glycogenolysis, you must draw the complete molecular structure of the conversion:

G-6-P → G-1-P (and vice versa).

For all subsequent steps in these pathways, you may write only the names of the molecules (no structures needed).

8. One Single Integrated Map – No Duplicate Molecules

All pathways must be presented in a single, unified map.

Do not repeat the same molecule unless it appears in different cellular compartments (e.g., cytosol, mitochondria, or peroxisome). In that case, repetition is allowed to reflect compartmentalization.

9. Compartment-Specific Placement

Any molecule or enzyme that participates in mitochondrial or peroxisomal processes must be placed inside a drawing that clearly represents the mitochondrion or peroxisome, respectively.

This visually shows the correct subcellular location.
