# Supplementary material for: Enhancing learning gains through gamification and collaborative learning strategies
Source: Front Med (Lausanne). 2026 Jul 31;13:1887898. doi: 10.3389/fmed.2026.1887898 (PMC13474011; doi:10.3389/fmed.2026.1887898)
Supplement: Supplementary file 2 [file Table_2.docx]

Supplementary file 2

**Final Metabolism Project: Metabolic Map**

As their final project, students will work in teams of 3 members, assigned by the professor, to create a metabolic map in poster format. The map must integrate the main metabolic pathways covered throughout the course. The following pathways must be included in your Metabolic Map:

- Glycolysis

- Gluconeogenesis

- Anaerobic fates of pyruvate

- Glycogenesis

- Glycogenolysis

- Non-oxidative phase of the Pentose Phosphate Pathway

- Oxidative phase of the Pentose Phosphate Pathway

- Fructose and galactose metabolism

- Synthesis of acetyl-CoA from pyruvate

- Krebs Cycle (Citric Acid Cycle)

- Anaplerotic reactions

- Electron Transport Chain

- Oxidative phosphorylation

- Activation and transport of fatty acids into the mitochondria

- Beta-oxidation of fatty acids (16-carbon)

- Ketone bodies

- Synthesis of 16-carbon fatty acids

- Origin of acetyl-CoA and NADPH for fatty acid synthesis

- *De novo* cholesterol synthesis (only major steps required)

- Amino acid degradation (indicate only the fate of carbon skeletons)

- Urea Cycle, including its connection to the Krebs Cycle and the diversion of aspartate-argininosuccinate

- Biosynthesis of each of the 20 amino acids (only indicate major precursors)

- Purine and pyrimidine degradation

- Synthesis of nitrogenous bases and nucleotide formation

Guidelines for Creating the Metabolic Map

1. Poster Format and Dimensions

The project must be presented as a white-background poster with the following dimensions:

Width: 1.10 to 1.60 meters

Length: 1.5 to 2.0 meters

2. Materials

You may choose the most appropriate materials to clearly illustrate the metabolic pathways. This includes colored pencils, different types of paper, or any other suitable supplies.

3. Hand-Drawn Only

The work must be entirely hand-drawn. Digital or printed maps are not permitted.

4. Readability

The poster must be legible from a distance of 1.5 meters.

5. Formal Attire

Students must dress formally during the presentation. Please bring a clean lab coat or jacket if you have one.

6. Required Content

The map must include the following:

I. Substrates, Intermediates, and Products

- Names and chemical formulas of all substrates, intermediates, and final products.

II. Enzymes, Cofactors, and Regulation

- Names of all enzymes, cofactors, coenzymes, and **hormones** involved.

- Also include **inhibitors and stimulators at key regulatory points.**

III. Pathway Connection

- It is essential to connect pathways through common metabolites (such as glucose-6-phosphate, pyruvate, acetyl-CoA, etc.).

- Do not repeat metabolites elsewhere on the map.

- The only exception is when the same metabolite appears in different cellular compartments (e.g., mitochondria, peroxisome, and cytoplasm).

- In each compartment, the metabolite may appear only once.

IV. Creativity and Innovation

- Apply creative and innovative ideas to make your map visually clear, organized, and engaging.

7. Grading Criteria

- There will be a team grade based on the overall quality of the map and each member's participation percentage.

- Additionally, each student will receive an individual grade (see the evaluation rubric).

- All team members must actively participate in the preparation of the map. Students who do not contribute will receive no credit for this portion of the grade.
