# Supplementary material for: Enhancing learning gains through gamification and collaborative learning strategies
Source: Front Med (Lausanne). 2026 Jul 31;13:1887898. doi: 10.3389/fmed.2026.1887898 (PMC13474011; doi:10.3389/fmed.2026.1887898)
Supplement: Supplementary file 3 [file Table_3.docx]

Supplementary file 3

**Evaluation rubric metabolic map**

Name of the professor for the team presenting the map: ___________________ GROUP: _________ TEAM: _________

Name of the evaluating student ______________________________________________________________________

**Note: To receive a grade for Parts 1 and 2, students must obtain a minimum INDIVIDUAL PARTICIPATION score of 6 or above.**

**Part 1: Map quality**

| **Criteria** | **Description** | | | **Score** |
| --- | --- | --- | --- | --- |
| **Score range** | **0-5** | **6-8** | **9-10** |  |
| **Organization, Cleanliness, and Clarity** | The map is messy, hard to read, or disorganized. | The map is somewhat organized but has some flaws. | The map is neat, clean, and easy to follow. |  |
| **Visual Layout and Pathway Arrangement** | Pathways are confusing and difficult to locate. | Pathways show some logical arrangement but need improvement. | Pathways are excellently organized and clearly arranged. |  |
| **Creativity and Use of Materials** | No creative elements were used. | Some creativity is present. | Highly creative, original, and innovative use of materials. |  |
|  |  | **Total score**  **Maximal possible score: 30 points** | |  |

**Part 2: Contents checklist**

1. All metabolite and enzyme names and chemical structures are included.

2. All metabolites and enzymes are correctly placed in their cellular compartments.

3. Pathways are connected through shared metabolites without repetition (exception: same metabolite may appear once per compartment).

4. All cofactors, coenzymes, inhibitors, and stimulators are shown at regulatory points.

5. The students were able to clearly present the metabolic pathway to the reviewer and correctly identify all its components.

In this section, you will rate each item using the following scale:

**Scoring Summary**

**Score range	Description**

0	Not present / Missing

3	Presented but incomplete

4	Fully presented

5	Exceptional presentation

|  | **Score** |
| --- | --- |
| **Glycolysis, gluconeogenesis and anaerobic fates of pyruvate** |  |
| **Glycogenesis and glycogenolysis** |  |
| **Oxidative and non-oxidative phase of the pentose phosphate pathway** |  |
| **Fructose and galactose metabolism** |  |
| **Synthesis of acetyl-CoA from pyruvate and Krebs cycle (Citric acid cycle)** |  |
| **Electron Transport Chain and oxidative phosphorylation** |  |
| **Activation and transport of fatty acids into the mitochondria and β-oxidation of fatty acids** |  |
| **Ketone bodies metabolism** |  |
| **Malate-aspartate shuttle system and synthesis of fatty acids** |  |
| **Cholesterol synthesis (only major steps with required)** |  |
| **Amino acid degradation (indicate only the fate of carbon skeletons) and urea cycle** |  |
| **Purine and pyrimidine degradation and de novo nucleotide synthesis** |  |
| **Total score maximal possible score: 60 points** |  |

**INDIVIDUAL PARTICIPATION AND TEAM SCORE**

Names of the students from the presenting team:	Answered questions correctly

(**max score 10**), OR did not

participate AND if they did,

always answered incorrectly (0)

**Total score**

**Score	(Maximum: 100 points)**

1. ______________________________________	______________________	**+ Part 1 and 2**	______

2. ______________________________________	______________________	**+ Part 1 and 2**	______

3. ______________________________________	______________________	**+ Part 1 and 2**	______

**Team average score_________**

**Select one or two badges you would award to this team:**

|  | **Badge** | **How to Earn It** |
| --- | --- | --- |
|  | **Master Artist** | The map is visually attractive and aesthetically pleasing. |
|  | **Metabolic Overlord** | The map includes ALL required metabolic pathways. |
|  | **Enzyme Expert** | All enzymes, cofactors, and coenzymes are correctly named. |
|  | **Formal Pro** | All team members attend in formal attire and arrive on time. |
|  | **Most Original** | The map uses unusual, creative, or unexpected materials. |
|  | **Most Beautiful** | The map uses materials that reflect the presenters' identity or personal style (e.g., cultural elements, favorite colors, thematic designs). |

**Other**_______________________
