# Supplementary material for: Enhancing learning gains through gamification and collaborative learning strategies
Source: Front Med (Lausanne). 2026 Jul 31;13:1887898. doi: 10.3389/fmed.2026.1887898 (PMC13474011; doi:10.3389/fmed.2026.1887898)
Supplement: Supplementary file 4 [file Table_4.docx]

Supplementary file 4

**Metabolic Map Competition – Evaluation Rubric**

General Rules

The top 10 highest-scoring teams will be selected from the competition.

In the event of a tie, all tied teams will be included in the selection.

Oral Evaluation Component

During the evaluation, the team will be asked 4 questions about the hormonal regulation of the different metabolic pathways or processes presented on their map.

Hormones to Be Covered

Insulin

Glucagon

Cortisol

Epinephrine (adrenaline)

Evaluation Procedure

Each team will be randomly assigned two hormones by the evaluators.

For each hormone, the team will be asked to identify a specific metabolic pathway or process affected by that hormone:

1. Explain the points of intervention (where and how the hormone acts).
2. Describe the effects of the hormone on the pathway (stimulation, inhibition, or modulation).

**Scoring Criteria for Oral Evaluation**

| **Criteria** | **Oustanding**  **(5-4)** | **Satisfactory (3)** | | **Needs Improvement**  **(2-1)** | |  |
| --- | --- | --- | --- | --- | --- | --- |
| **Identification of the pathway** | Correctly identifies the pathway and its role. | | Identifies the pathway but with minor errors. | | Unable to identify the correct pathway. | |
| **Points of intervention** | Accurately describes all key regulatory points and enzymes targeted by the hormone. | | Describes some points but misses key details. | | Fails to describe any intervention points. | |
| **Effects of the hormone** | Clearly explains the hormonal effects (activation/inhibition) and their metabolic consequences. | | Explains effects partially or with some confusion. | | Unable to explain hormonal effects. | |
| **Clarity and coherence** | Explanation is clear, logical, and well-structured. | | Explanation is somewhat unclear or disorganized. | | Explanation is confusing or incoherent. | |

**Maximum Possible Score, 20 points**
