# Supplementary material for: Enhancing learning gains through gamification and collaborative learning strategies
Source: Front Med (Lausanne). 2026 Jul 31;13:1887898. doi: 10.3389/fmed.2026.1887898 (PMC13474011; doi:10.3389/fmed.2026.1887898)
Supplement: Supplementary file 5 [file Table_5.docx]

Supplementary file 5

True-false questions used to assess learning gain, the number of true responses is equivalent to false responses:

1. In metabolic pathways, enzymes that catalyze irreversible reactions are potential control points.
2. In glycolysis, the control points are the reactions catalyzed by phosphofructokinase, aldolase, and pyruvate kinase.
3. Glyceraldehyde-3-phosphate dehydrogenase is the main control point of glycolysis.
4. Phosphofructokinase-1 is inhibited by high [ATP] because it decreases its affinity for fructose 6-phosphate.
5. Phosphorylation of glycogen phosphorylase activates the breakdown of glycogen.
6. A carbon leaves the Krebs cycle in the form of CO_2_ through the oxidation of isocitrate and alpha-ketoglutarate.
7. In the conversion of succinyl-CoA to succinate, one GTP is hydrolyzed to GDP.
8. The lactate formed mainly in the muscle is released into the blood and transported to the intestine where it is metabolized back to glucose.
9. In aerobic organisms, the citric acid cycle is an important source of energy, so replenishment reactions known as anaplerotic reactions are necessary to restore the intermediates of the cycle.
10. The presence of high cytosolic concentrations of citrate in the presence of ATP causes the accumulation of glucose 6-P and fructose 6-P.
11. The accumulation of fructose 2,6-bisphosphate activates hexokinase.
12. In the Cori cycle, glucose is released from the muscle into the bloodstream and transported to the liver.
13. The increase in mitochondrial Ca^2+^ concentration inhibits the citric acid cycle.
14. An important pathway where NADPH is produced is the oxidative phase of the pentose phosphate pathway.
15. Uridine diphosphate glucose (UDP-glucose or UDPG) is the activate glucose donor required for glycogen synthesis.
16. Fatty acids are stored as triglycerides in adipocytes.
17. Before β-oxidation, long-chain fatty acids are activated to acyl-CoA in the cytosol, then acyl groups are transferred to carnitine via carnitine palmitoyltransferase I to form acyl-carnitine for mitochondrial import.
18. In β-oxidation, ATP, NADH, and FADH2 are consumed to degrade amino acids.
19. Ketone bodies are synthesized in the peroxisome.
20. The first step in fatty acid synthesis is the formation of malate.
21. β-oxidation involves the sequential removal of two-carbon units from the fatty acid chain.
22. β-Hydroxy β-methylglutaryl-CoA reductase is the most regulated enzyme in cholesterol synthesis.
23. Chylomicron are very large lipoproteins that primarily transport triacylglycerols from food from the intestine.
24. Peripheral tissues can transport nitrogen to the liver, where it is converted into urea.
25. Acetyl-CoA is essential in the urea cycle because it is the second nitrogen donor.
26. Lactate participates in the Cori cycle.
27. Enolase participates in both glycolysis and gluconeogenesis.
28. Acyl-CoA is the main substrate in cholesterol synthesis.
29. Xylulose-5-phosphate is an intermediate in the non-oxidative phase of the pentose phosphate pathway.
30. Trypsin hydrolyzes proteins at their aromatic residues.
31. Glutamate donates amino groups to pyruvate in the Cori cycle.
32. α-Ketoglutarate is an intermediate in amino acid degradation and the citric acid cycle.
33. The regulation of the urea cycle is controlled by carbamoyl-phosphate synthetase I.
34. Glycine is a ketogenic amino acid.
35. Glutamate is an amino acid that forms α-ketoglutarate exclusively through its anabolic pathway.
36. All the nitrogen necessary to generate *de novo* pyrimidines comes from amino acids.
37. Vitamin B12 is necessary for the *de novo* synthesis of purines.
38. Carbamoyl-phosphate synthetase II is involved in nucleotide synthesis.
39. Ribonucleotide reductase participates in the formation of ribonucleotides.
40. Nucleotide synthesis requires the involvement of various precursors, including fatty acids.
